# Antimicrobial action and anti-corrosion effect against sulfate reducing bacteria by lemongrass (*Cymbopogon citratus*) essential oil and its major component, the citral

**DOI:** 10.1186/2191-0855-3-44

**Published:** 2013-08-10

**Authors:** Elisa Korenblum, Fátima Regina de Vasconcelos Goulart, Igor de Almeida Rodrigues, Fernanda Abreu, Ulysses Lins, Péricles Barreto Alves, Arie Fitzgerald Blank, Érika Valoni, Gina V Sebastián, Daniela Sales Alviano, Celuta Sales Alviano, Lucy Seldin

**Affiliations:** 1 Departamento de Microbiologia Geral, Instituto de Microbiologia Paulo de Góes, Universidade Federal do Rio de Janeiro, Rio de Janeiro, Brazil; 2 Departamento de Engenharia Agronômica, Universidade Federal de Sergipe, Aracajú, SE, Brazil; 3 CENPES, Petrobras, Ilha do Fundão, Rio de Janeiro, Brazil

**Keywords:** Lemon grass essential oil, Citral, Antimicrobial activity, Anti-corrosion, Souring prevention, Sulfate reducing bacteria

## Abstract

The anti-corrosion effect and the antimicrobial activity of lemongrass essential oil (LEO) against the planktonic and sessile growth of a sulfate reducing bacterium (SRB) were evaluated. Minimum inhibitory concentration (MIC) of LEO and its major component, the citral, was 0.17 mg ml^-1^. In addition, both LEO and citral showed an immediate killing effect against SRB in liquid medium, suggesting that citral is responsible for the antimicrobial activity of LEO against SRB. Transmission electron microscopy revealed that the MIC of LEO caused discernible cell membrane alterations and formed electron-dense inclusions. Neither biofilm formation nor corrosion was observed on carbon steel coupons after LEO treatment. LEO was effective for the control of the planktonic and sessile SRB growth and for the protection of carbon steel coupons against biocorrosion. The application of LEO as a potential biocide for SRB growth control in petroleum reservoirs and, consequently, for souring prevention, and/or as a coating protection against biocorrosion is of great interest for the petroleum industries.

## Introduction

Hydrocarbons in petroleum may serve as electron donors for sulfate reducing bacteria (SRB), which use sulfate as the terminal electron acceptor for respiration, resulting in sulfide production. The biogenic sulfide production results in metal biocorrosion and reservoir souring, and SRB are typically the main bacterial group involved in these harmful processes in petroleum industries. The biogenic hydrogen sulfide production causes the acidulation and plugging of petroleum reservoirs and biocorrosion of metal surfaces of pipelines and tanks (Nemati et al. 2001a). Moreover, the sulfide is explosive in high concentrations. SRB may grow in pipes and tanks forming biofilms, leading to the biodegradation of the metal surface (Zuo 2007). Finally, the accumulation of SRB biomass causes reduced oil recovery (Muyzer and Stams 2008; Nemati et al. 2001b; Postgate 1965). Therefore, in petroleum industries, it is mandatory to control and inhibit SRB growth, which is usually done by biocide dosage (Korenblum et al. 2010; Videla 2002). Regardless of the effectiveness of these biocides, antimicrobial resistance often occurs, particularly in biocide-treated biofilms (Fraise, 2002; Stewart and Costerton, 2001). In addition, the residual concentration, toxicity and persistence of biocides in industrial effluents is of high environmental concern. Hence, alternatives for SRB control are of great interest to the petroleum industry (Nemati et al. 2001; Stewart 2002).

Less expensive and environmental friendly treatments are sought by the petroleum industry as alternatives to the use of synthetic biocides. Essential oils are mixtures of lipophilic and volatile substances, which are known to have components with antibacterial and/or antifungal activity and are potential sources of novel inhibitory substances (Hammer et al. 1999; Solórzano-Santos and Miranda-Novales 2012). The composition of essentials oils is different among species and plant parts. The oil’s main components are terpenes and terpenoids, which are aromatic and aliphatic acid esters and phenolic compounds (Reichling et al. 2009). The effect of different plant extracts on biofilms has already been demonstrated in the food industry and medical devices. In addition, unlike other natural antimicrobial compounds, essential oils show inhibition on planktonic and sessile microbial growth at the same concentration. Thus, the ability to form biofilms does not provide extra protection for the organism when using essential oils as an antimicrobial agent (Adukwu et al. 2012; Kavanaugh and Ribbeck 2012; Nostro et al. 2007; Nuryastuti et al. 2009). Citral, the principal compound of lemongrass (*Cymbopogon citratus* (DC.) Stapf) essential oil (LEO), is valued as an antimicrobial compound against several important medical and food bacteria, such as *Campylobacter jejuni*, *Escherichia coli* O157, *Listeria monocytogenes*, *Bacillus cereus* and *Staphylococcus aureus* (Fisher and Phillips 2006). The citral is found at 65-85% of total compounds in LEO as two evenly distributed isomers, neral and geranial (Moore-Neibel et al. 2012). In Brazil, lemongrass essential oil is constituted of up to 75.4% of citral (Barbosa et al. 2008).

In this study, for the first time, the use of LEO or citral are being proposed for application by the petroleum industry to control and/or remove SRB biofilm formation and sulfide induced corrosion of metal surfaces, such as in pipes and tanks. For this purpose, we have tested the lemongrass essential oil and a commercial citral against the SRB *Desulfovibrio alaskensis* strain NCIMB 13491 during planktonic growth and biofilm development on glass and carbon steel coupons.

## Materials and methods

### Plant essential oil isolation and citral

The lemongrass (*Cymbopogon citratus* (DC.) Stapf, *Poaceae*) leaves were collected from the medicinal plant garden of the Federal University of Sergipe, Brazil. A voucher specimen was deposited at the Herbarium-ASE of the Federal University of Sergipe (code 9391). Lemongrass essential oil (LEO) was obtained from the fresh leaves by hydrodistillation using a glass-type *Clevenger* apparatus continuously for 8 h and stored in an opaque glass vial at 4°C prior to analysis and biological assays. LEO (*d* = 0.85 g ml^-1^) was dissolved in Postgate medium for all experiments, at desired concentrations. The commercial citral (>96% purity) used was purchased from Sigma-Aldrich (Castle Hill, NSW, Australia) and used without further purification.

### Sulfate reducing bacterial strain

The test microorganism used in this study was *Desulfovibrio alaskensis* NCIMB 13491. This SRB strain was isolated from a soured oil reservoir (Feio et al. 2004) and was usually grown in Postgate C or Postgate E media (Postgate 1984) at 30°C for 3 days, in anaerobic conditions using sealed serum bottles (10 ml). The bottles were purged with a N_2_ flux to achieve anaerobiosis.

### Determination of the minimum inhibitory concentration (MIC) and minimum bactericidal concentration (MBC)

In order to establish the minimum concentration that the LEO and citral inhibit *D*. *alaskensis* NCIMB 13491 growth, microdilution susceptibility tests were performed (Das et al. 2008). The working solutions of LEO (0.5 mg ml^-1^) and citral (1.0 mg ml^-1^) were serially diluted in 96-well microtiter plates, to a lowest concentration of 0.085 mg ml^-1^ in sterile Postgate medium to determine the minimum inhibitory and the minimum bactericidal concentrations. The indicator strain *D*. *alaskensis* was grown for 7 days at 32°C in Postgate E medium. This culture was further diluted to yield a final SRB inoculum of 10^5^ cells ml^-1^. The microtiter plates were incubated for 7 days at 32°C. The *D*. *alaskensis* growth was detected by observing the blackish color of the medium caused by iron sulfide precipitation in Postgate E medium. The minimum inhibitory concentration (MIC) was determined as the least amount of antimicrobial substance added that did not result in blackish color of the medium. Sub-MIC (0.5× MIC) and supra-MIC (2× MIC) of LEO were also established and used for further tests (Transmission microscopy and biofilm assays). To perform the minimum bactericidal concentration test, aliquots of 10 μl of the treated and untreated cell suspensions from the MIC plate were used to inoculate fresh Postgate E medium (90 μl) and then incubated for 7 days at 32°C. The minimum bactericidal concentration (MBC) was determined as the lowest concentration of antimicrobial substance that resulted in no growth of *D*. *alaskensis* indicator strain. All of the inoculation procedures and incubations were performed in an anaerobic chamber (PlasLabs Inc., USA). The time kill method was also performed using LEO and citral macrodilutions in Postgate E broth (2 ml) in BD Vacutainer™ tubes, where 10^5^ SRB cells ml^-1^ were later inoculated. The MIC levels of LEO and of citral were tested by incubating Vacutainer tubes for 0.5, 2, 6, 12 and 24 hours at 32°C. In addition, one test tube was not incubated, and cells were only rapidly homogenized with LEO or citral. An untreated sample was collected as a control for all incubation times. After incubation, biomass was recovered from Vacutainer tubes by centrifugation and further cell pellets were washed with N_2_ purged sterile distilled water twice to remove broth and LEO or citral residues. Then, each cell pellet was suspended in fresh Postgate E medium (transferred with a syringe into the Vacutainer tube) and incubated for 7 days at 32°C. All tests were performed in five replicates.

### Real-time PCR analysis

DNA was extracted from 10 ml of each sample of *D*. *alaskensis* treated with MIC, sub-MIC and supra-MIC of LEO and citral. Samples were centrifuged for 20 min at 12.800 ×g. The pellets were suspended in 500 μl of TE 1× and then the extraction was performed as described by Pitcher et al. (1989). Real-time PCR assay of *dsr*A gene (encodes the α subunit of dissimilatory sulfite reductase) was performed as described previously (Spence et al. 2008) for detection and enumeration of sulfate-reducing bacteria, on an Applied BioSystems Instrument (Life Technologies, SP, Brazil) using the SYBR Green PCR kit (Qiagen). An aliquot (5 μl) of template DNA was used in a reaction mixture containing 10 μl 2× Quanti Tect SYBR Green PCR Master Mix, 500 nmol l^-1^ of each forward and reverse *dsrA* primer (Dsr1f – 5ACSCACTGGAAGCACGGCGG3’; DsrR– 5’GTGGMRCCGTGCAKRTTGG3’), and water to a final volume of 20 μl. Reaction conditions were 95°C for 15 min (1×), then 95°C 15 s, 59°C 30 s, 72°C 30 s (40×). A final melt curve analysis was performed to determine the presence or absence of non-specific amplification products.

### Preparation of cells for transmission electron microscopy (TEM)

For TEM characterization of the biocidal effect of LEO on *D*. *alaskensis* cells, *D*. *alaskensis* was inoculated at 10^5^ bacterial cells ml^-1^ with LEO (at MIC, sub-MIC and supra-MIC) at 30°C for 24 hours. In addition to the treated cells, *D*. *alaskensis* without LEO was incubated at the same conditions as a control sample. The cells were washed in PBS buffer and fixed overnight at 4°C in 2.5% glutaraldehyde in 0.1 M sodium cacodylate buffer. Samples were washed three times in the same buffer, post-fixed at room temperature in 1% osmium tetroxide in 0.1 M sodium cacodylate buffer, washed three times in the same buffer, dehydrated in an acetone series and embedded in Polybed 812 resin. Ultra-thin sections were obtained using a Leica ultramicrotome and stained with uranyl acetate and lead citrate. Imaging was done using a FEI Morgagni transmission electron microscope at 80 kV. Treated control samples were prepared in duplicate.

### Antimicrobial action of LEO on SRB biofilm formation and stability on glass coupon

Biofilms of *D*. *alaskensis* were prepared by inoculating circular cover glass slides with a mid-log phase culture (10^5^ cells ml^-1^) grown in Posgate E medium. Before each experiment, the surface of the glass cover slides (13 mm diameter and 1.2 mm thick) was treated with a cleaning solution (Korenblum et al. 2008). The device for biofilm formation was a 24-well-plate with a cover glass slide in each well. Two assays were used to test the efficacy of the LEO against SRB biofilm formation. For the two assays, LEO at MIC, sub-MIC and supra-MIC concentrations was added as follows: (assay 1) LEO added at the same time that the SRB cells were introduced in the biofilm device; and (assay 2) planktonic SRB cells were incubated with LEO for 24 h and then these pretreated cells were introduced in the biofilm system. A control without LEO was performed in both assays. Two milliliters of the cell suspension (pre-treated with LEO, or untreated) was added to each well, covering the glass surfaces, and incubated at 30°C for 7 days. After the incubation period for biofilm formation on the glass surfaces, unattached or loosely attached cells were removed by washing with N_2_ purged distilled water, prior to biofilm cell enumeration. Biofilm cells were enumerated (30 fields) using counter-staining with 10 μg ml^-1^ DAPI (diamidino-2-phenylindole) to determine total cells counts and 100 μg ml^-1^ propidium iodide, for dead cells counts, using a fluorescence microscope (Zeiss Axioplan 2). This procedure was repeated three times for each coupon. The results were expressed as the mean value, and standard deviation as required.

### Conditioning carbon steel coupons with LEO

Carbon steel coupon (20 mm×10 mm×2 mm) surfaces were treated using a sandblasting technique and were cleaned prior to the biofilm assay, as described previously (Nemati et al. 2001b). Briefly, the coupons were cleaned in 18% HCl, which was then neutralized by immersion in a saturated sodium bicarbonate solution. Finally, coupons were washed with distilled water, rinsed in acetone, and dried in an air stream. Conditioned surfaces were obtained by immersion of carbon steel coupons in LEO at MIC, sub-MIC and supra-MIC solutions for 24 h at 20°C. A cell control (without LEO) and a blank control (without cells) were performed using untreated coupons. Treated and control coupons were performed in 4 replicates. The remaining amount of LEO that was not absorbed to the metal surface was then removed by rinsing with deionised water and dried with sterile air. The cleaned coupons were placed in tubes containing Postgate C medium inoculated with 10^5^ cells ml^-1^ of *D*. *alaskensis* and incubated for 7 days. The effect of LEO was evaluated under macroscopic observations. In addition, to analyze coupon weight loss coupon surfaces were cleaned (washed in acid, neutralized with sodium bicarbonate, rinsed in water and acetone, and dried in an air stream) and coupon weight loss was determined by measuring the weight of the coupon using an analytical balance (Sartorius AG model TE214S, Goettingen, Germany) as previously described (Marques et al. 2012). Weight was measured in grams, and the corrosion rate (CR) of carbon steel coupons was calculated and is expressed in mm year^-1^ (ASTM G4/95 2001), using 7.84 g cm^-3^ as the density of carbon steel. The average corrosion observed on blank coupons was subtracted from cell control (untreated) and conditioned coupons weight loss values. A two-sample *t* test was performed on treated and control coupons.

## Results

**Figure 1:**
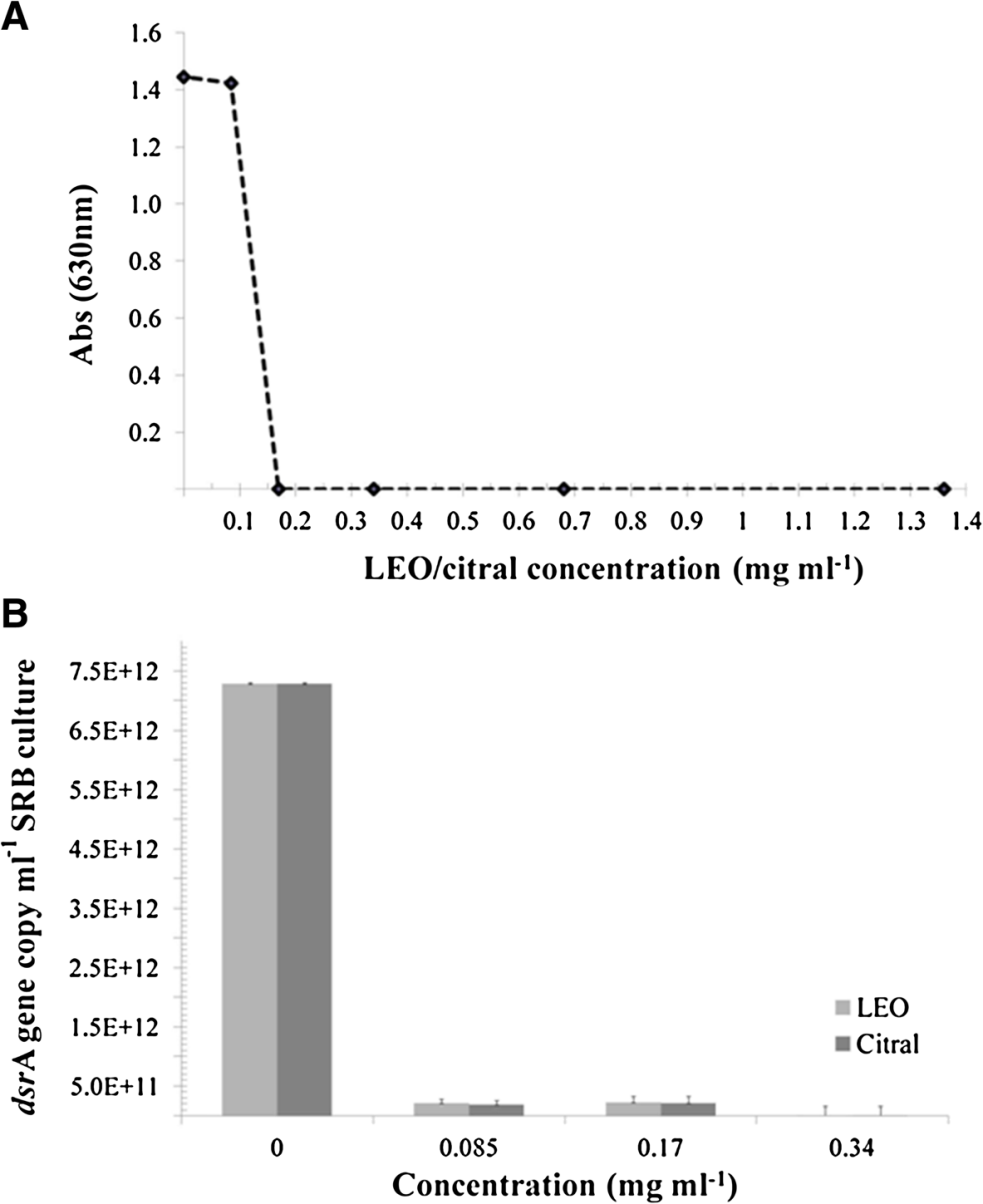
**Determination of the MIC of LEO and citral using optical densities of cell cultures (A), and *dsrA *gene copy (B).**

Lemongrass essential oil (LEO) and its major constituent – citral were tested against a SRB strain (*D*. *alaskensis* NCIMB 13491) in planktonic and sessile growth stages. The minimum inhibitory concentration (MIC) of LEO and citral were determined by measuring the optical density of *D*. *alaskensis* culture and by real time PCR using the *dsrA* gene, which encodes the α subunit of a key enzyme of SRB metabolism, the dissimilatory sulfite reductase. The MIC of either LEO or citral was established at 0.17 mg ml^-1^ according to the spectrophotometric assay result (Figure 1A), while the real time PCR of *dsrA* gene indicated a MIC of 0.085 mg ml^-1^ (Figure 1B). Thus, citral was evinced to be responsible for the antimicrobial effect in LEO, as no inhibition difference was observed between the essential oil and its main component. In order to proceed with the following tests with a strict threshold levels, MIC, sub-MIC and supra-MIC levels were considered as 0.17 mg ml^-1^, 0.085 mg ml^-1^ and 0.34 mg ml^-1^, respectively. The minimum bactericidal concentration (MBC) of LEO or citral was determined as the same value as the MIC, as no cell growth was recovered from any of the five replicate wells. Therefore, LEO showed a bactericidal effect against the SRB *D*. *alaskensis*. The time kill test at MIC level showed an immediate bactericidal effect, that is, no viable cells were recovered after SRB growth in Postgate E medium.

**Figure 2:**
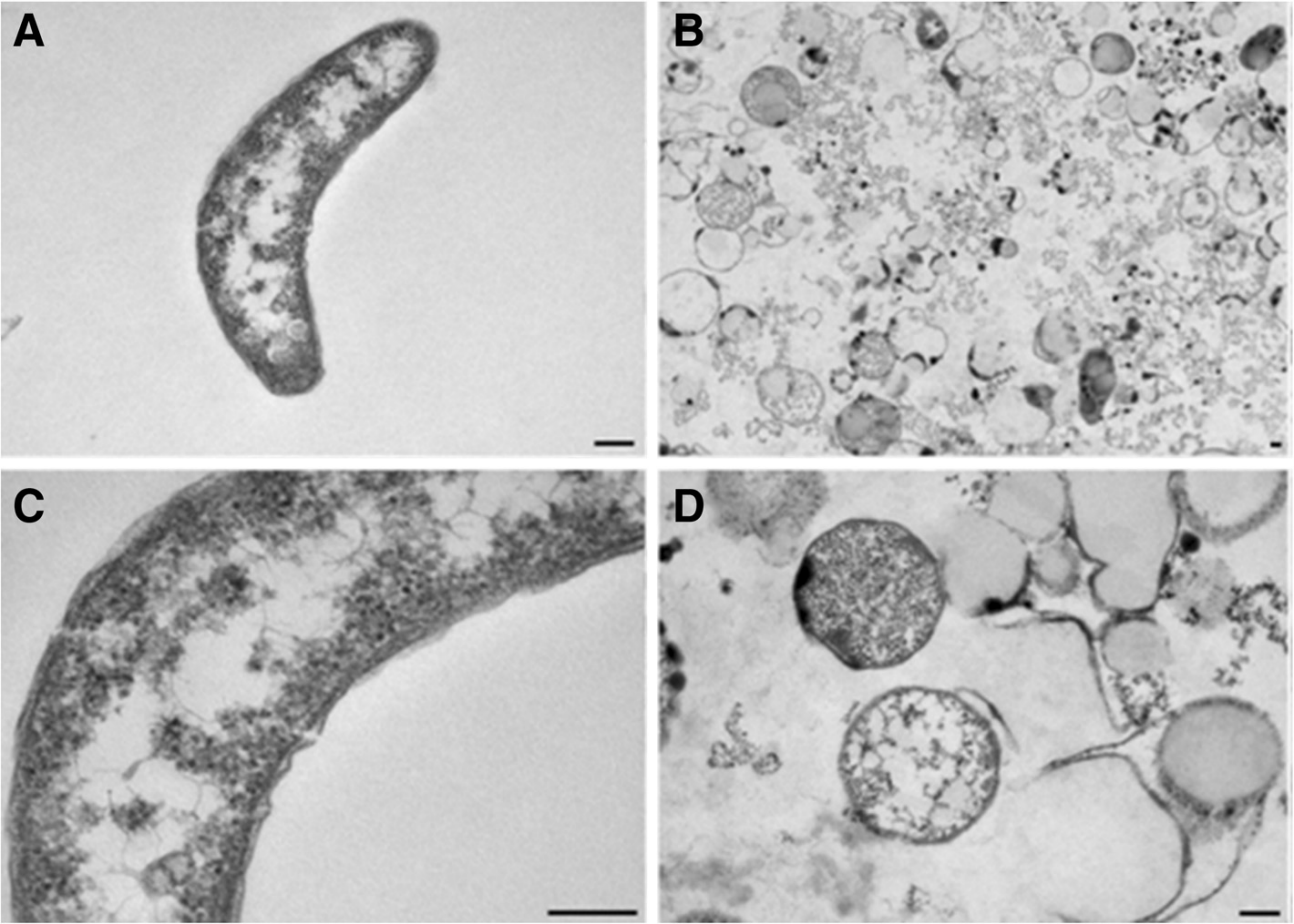
**Transmission electron micrographs of non-treated (A and B) and LEO-treated (C and D) *Desulfovibrio alaskensis *cells.** Scale bars indicate 200 nm in all images.

**Figure 3:**
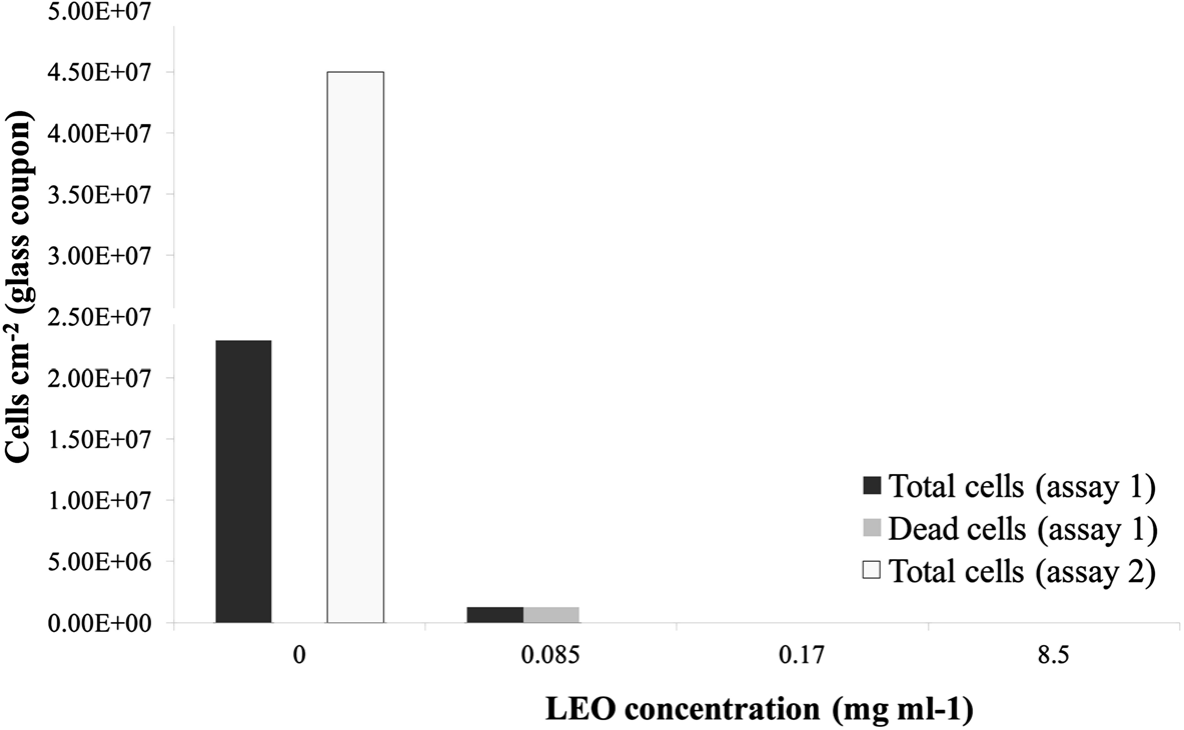
Activity of LEO on SRB cells, where LEO was introduced together with cells in the biofilm device (assay 1), and LEO pre-treated SRB cells inhibited biofilm formation on glass surfaces (assay 2).

**Figure 4:**
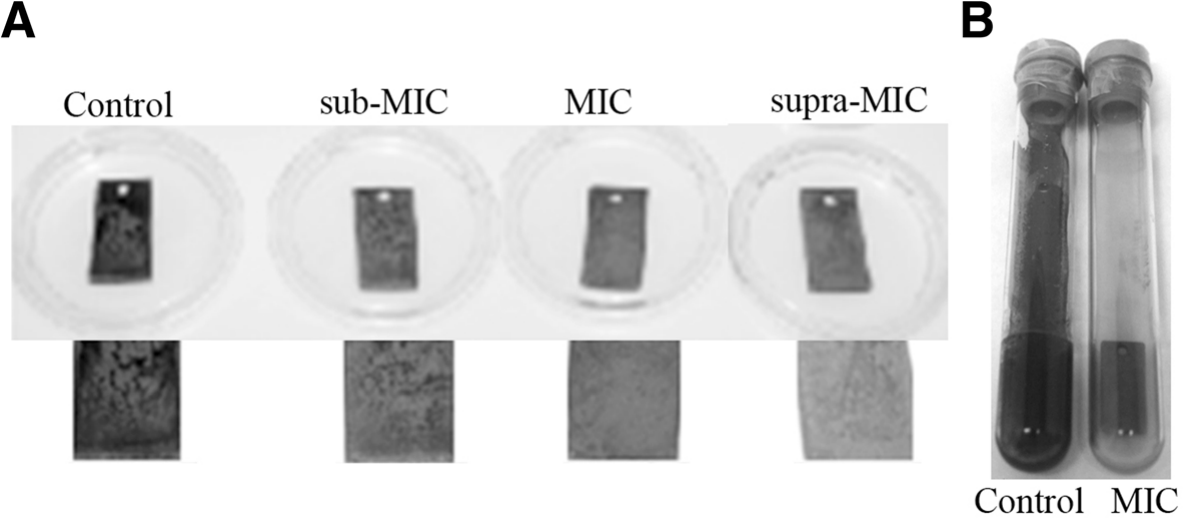
SRB biofilm formation on control coupon and inhibition of biofilm on coupons conditioned with LEO at MIC, sub-MIC and supra-MIC solutions for 24 h at 20°C (A), experiment performed in BD Vacutainer tubes; control coupon and conditioned coupon with LEO at MIC (B).

The ultrastructure of untreated *D*. *alaskensis* showed cells with a vibrio-shaped morphology (Figure 2A) and regular Gram-negative double membrane layered cell wall structure (Figure 2B). The cytoplasm was electron-translucent and contained no conspicuous reserve granules. Cells treated with a MIC level (0.17 mg ml^-1^) of LEO revealed strong morphological and cytoplasmatic alterations (Figure 2C). Cytoplasm leakage was observed in many cells suggesting loss of cell constituents and lysis caused by LEO treatment (Figure 2C and D). Despite the fact that a few treated cells presented a cytoplasmatic appearance similar to the control, unusual electron-dense granules near the cell membrane could be observed (Figure 2D). The indication of cell morphology alterations after the LEO treatment, especially lysis, corroborates its bactericidal effect. *D*. *alaskensis* biofilm removal (assay 1) and sessile growth inhibition (assay 2) by LEO was observed when testing biofilm formation on glass coupons (Figure 3). The essential oil was a highly effective biofilm inhibitor. In assay 1, LEO killed all the SRB cells at MIC (0.17 mg ml^-1^) and supra-MIC (0.34 mg ml^-1^) levels. Sub-MIC level (0.085 mg ml^-1^) showed one log reduction of SRB cells; however, adhered cells left on the surface were dead, as detected by propidium iodide staining. In addition, in assay 2, LEO inhibited SRB biofilm formation, when planktonic cells were pretreated with sub-MIC, MIC and supra-MIC values. Untreated SRB cells formed biofilms on glass surfaces in both assays (10^7^ cells cm^-2^) (Figure 4). The inhibition of SRB biofilm formation was also observed on carbon steel coupons conditioned with LEO (Figure 4). The black corrosion precipitates could be observed in the control carbon steel coupons, while the treated coupons were preserved from biofilm formation and therefore from SRB-induced biocorrosion processes. There was a slight corrosion detected on conditioned coupons, it was very similar to the corrosion observed on blank coupons (p=0.54), which was considered as chemical corrosion. Weight loss of the untreated coupons was significantly higher than on conditioned coupons, when blank coupons corrosion rate was subtracted. Then, there was no biocorrosion on conditioned coupons, while a high biocorrosion rate was detected on untreated ones (1.06 (±0.1) 10^7^ mm year^-1^, p<0.01).

## Discussion

In this study, SRB planktonic and sessile growth was shown to be inhibited by lemongrass essential oil (LEO) and its major component, the citral. Different essential oils of plants have been shown to have an antimicrobial activity (Abo-El Seoud et al. 2005; Bajpai et al. 2009; Bakkali et al. 2008; Khan and Ahmad 2011). In medicine, essential oils extracted from plants present antimicrobial activity against a range of bacteria including known antibiotic resistant strains (i.e. MRSA) and have been used as topical and oral antimicrobial treatments (Doran et al. 2009; Solórzano-Santos and Miranda-Novales , Solórzano-Santos and Miranda-Novales Solórzano-Santos and Miranda-Novales 2012). Moreover, the inhibitory activity of essential oils has already been demonstrated to be effective against biofilms formed by bacteria of medical relevance (Adukwu et al. 2012; Khan et al. 2009). Adukwu et al. (2012) have screened the antimicrobial activity of different essential oils against *Staphylococcus aureus* strains and they found LEO to be the most effective oil.

Two tests were performed to determine MIC of LEO, each test indicated different values, cell culture absorbance (0.17 mg ml^-1^) and real time PCR of the bacterial *dsrA* gene (0.085 mg ml^-1^). Both tests were also performed with citral, and the MIC results were identical to LEO. In order to set meticulously the inhibitory concentration level, MIC value of LEO and of citral, the predominant terpene of LEO, was considered to be 0.17 mg ml^-1^ for the inhibition of SB growth. The MIC value of LEO in the present study corresponds to 0.1% (v/v), likewise, Adukwu et al. (2012) and Doran et al. (2009) reported LEO to be effective against other bacteria at MIC between 0.06 and 0.16% (v/v).

Ultrastructural changes were observed when *D*. *alaskensis* was treated with LEO. Electron microscopy showed that most of the cells either presented cytoplasmatic extraction or were completely disrupted, suggesting that LEO provokes harsh membrane disturbance. In addition, electron-dense granules near the cytoplasmatic membrane could be observed in the remaining cells, indicating that LEO treatment also influenced cellular metabolism. A previous study had also observed that essential oils alter bacterial membrane stability and lead to the loss of cytoplasmatic material (Bouhdid et al. 2010). Essential oils present lipophilic properties that allow it to pass through the bacterial cell wall. Cell wall disruption occurs at different layers of polysaccharides, fatty acids and phospholipids (Fadli et al. 2012). The ultimate effect of essential oils is cell death due to permeabilization of the cellular membrane, loss of ions, reduction of membrane potential, collapse of the proton pump and depletion of the ATP pool (Bakkali et al. 2008).

The hindrance of SRB cell attachment on glass and metal surfaces, and the ability to remove pre-established biofilms was also seen when LEO was applied at sub-MIC, MIC and supra-MIC levels. Although the effect of essential oils on biofilms has already been reported in for medically important bacteria, this is the first study that shows the antimicrobial activity of an essential oil against anaerobic bacteria of relevance to the petroleum industry. In addition, LEO was shown to prevent biocorrosion (weight loss) of carbon steel coupons by inhibiting the sulfate-reducing bacterium *Desulfovibrio alaskensis* biofilm formation. Biofilms are an agglomeration of microbial cells that adhere to a surface and are imbibed in a polymeric matrix built by the microorganisms themselves (Costerton 1999). This structure changes the physiological state when compared to their planktonic counterparts, and then these sessile cells have a better fitness in natural environments (Golby et al. 2012). In petroleum environments, SRB biofilms have been associated with biocorrosion of metal surfaces of the petroleum production line (Jayaraman et al. 1999). Therefore, the use of LEO, or citral, at MIC and supra-MIC levels to control SRB biofilms fits the golden rule that should be applied to all industrial systems described by (Videla et al. 2002), which is to keep the system clean in order to avoid biocorrosion.

Our findings showed that the essential oil of lemongrass has antimicrobial activity against SRB and anti-biocorrosion effect on carbon steel metal. Besides that, the main component of LEO, citral, which is an oxygenated terpene, has shown an active inhibition of SRB growth (this study) and other bacteria (Reichling et al. 2009; Solórzano-Santos and Miranda-Novales , Solórzano-Santos and Miranda-Novales Solórzano-Santos and Miranda-Novales 2012). Our findings showed that the LEO has antimicrobial activity against SRB growth and controls biocorrosion on carbon steel metal. In petroleum industries, LEO and citral may be used in formulations in the same manner as synthetic biocides; and may be formulated in water, methanol or isopropanol. We propose that LEO and citral antimicrobial activity and the consequent anti-corrosion effect are a future option to control SRB planktonic and sessile growth, as well as biocorrosion mitigation in petroleum industrial facilities.

## Competing interests

The authors declare that they have no competing interests.
